# Routing Protocol for Underwater Wireless Sensor Networks Based on a Trust Model and Void-Avoided Algorithm

**DOI:** 10.3390/s24237614

**Published:** 2024-11-28

**Authors:** Jun Ye, Weili Jiang

**Affiliations:** Key Laboratory of Internet Information Retrieval of Hainan Province, School of Cyberspace Security, Hainan University, 58 Renmin Avenue, Haikou 570228, China; jiangweili@hainanu.edu.cn

**Keywords:** trust model, secure transmission, fuzzy logic, underwater wireless sensor networks

## Abstract

Underwater wireless sensor networks have a wide range of application prospects in important fields such as ocean exploration and underwater environment monitoring. However, the influence of complex underwater environments makes underwater wireless sensor networks subject to many limitations, such as resource limitation, channel openness, malicious attacks, and other problems. To address the above issues, we propose a routing scheme for underwater wireless networks based on a trust model and Void-Avoided algorithm. The proposed scheme establishes a trust model, evaluates the behavior of underwater nodes through direct trust, indirect trust, and environmental trust, and finds malicious nodes while taking into account evaluation of the channel, which provides support for the next data transmission event. The proposed scheme prioritizes the total cabling distance and introduces a two-hop availability checking model for data transmission, checking the nodes for voids and avoiding the void areas, to find the transmission path with the lowest energy consumption and lowest latency as much as possible. In this study, simulation experiments were conducted on the proposed scheme, and the results showed that the target scheme can effectively detect malicious nodes through anomalous behaviors and outperforms existing work in terms of malicious node detection rate, energy consumption, and end-to-end latency, and network performance.

## 1. Introduction

Underwater Wireless Sensor Network (UWSN) technology is receiving widespread attention both at home and abroad and is being widely used for marine data collection, pollution prediction, offshore mining, shipwreck avoidance marine monitoring, and more. UWSNs are one of the key basic support technologies for the development of intelligent water conservancy and ocean exploration, and have broad application prospects in real-time water quality monitoring, water pollution control, and other aspects. However, UWSNs have many limitations compared with wireless sensor networks due to the specific conditions:

- Acoustic communication: Due to the large attenuation of electromagnetic waves and light waves underwater, hydroacoustic communication is the main means of underwater long-distance communication [1].
- Bandwidth: Acoustic waves travel underwater at 1500 m/s, which results in much lower bandwidth for acoustic communications compared to terrestrial wireless sensors (TWSNs) [2].
- High BER: Hydroacoustic channels are characterized by high time variation, long propagation delay, high signal attenuation, high environmental noise, and low available bandwidth, which, to a certain extent, result in high BER and high packet loss, affecting the efficiency and accuracy of data transmission [3].
- Environmental constraints: The harsh underwater environment constrains the computational resources and storage resources of UWSNs nodes. Underwater sensor nodes (UW-sensors) use batteries for their energy supply, and it is technically difficult to recharge the batteries of UW-sensors at a higher cost. In addition, due to environmental noise, channel fading, and so on, UW-sensors need to consume more energy to compensate for the signal during data transmission, which greatly increases the energy consumption of the UW-sensors [4].

All these factors converge to result in minimal resources available for UWSNs. They also limit the prospect of using some of the more mature security algorithms used in TWSNs in UWSNs. Due to the limited transmission range, UW-sensors usually relay the received data to the receiver nodes through multiple forwarding nodes, which provides conditions for malicious nodes (MNs) inside the network to damage the underwater wireless sensor network. MNs within the network can forge, discard, eavesdrop, and modify the transmitted data to affect the accuracy and efficiency of network data transmission. In addition to this, MNs can send a large number of useless and repetitive messages inside the network to occupy the network channel, consume a large amount of energy, and greatly reduce the survival time of the UWSNs. Numerous researchers have introduced trust models into UWSNs to identify MNs inside the network and drive them out of the network. This is a core element. The trust model assigns a trust value to each underwater sensor node, and other nodes confirm their trustworthiness before collaborating with them. The trust model can be applied to protocols such as routing protocols, localization, and synchronization to enhance the security of the UWSNs. Unlike authentication models, trust models usually work periodically to collect trust evidence and calculate the trustworthiness of nodes through their multifaceted performance. The trust model can effectively respond to internal attacks and improve network security.

We designed a new trust management scheme for UWSNs to ensure the security of packet transmission and improve the efficiency of data transmission. The main contributions of this paper are shown below:

- We propose a trust model for checking the trust performance of underwater nodes in UWSNs. The proposed scheme considers three metrics (environmental trust, direct trust, and indirect trust) to analyze the performance of UW-sensors, identify MNs, and find the set of forwarding nodes suitable for data transmission.
- The scheme proposed in this paper considers the total wiring distance and utilizes a two-hop availability checking algorithm to find the void nodes and improve the data transmission efficiency of the UWSNs.
- We evaluated the performance of the proposed scheme with HH-VBF [5], GEDAR [6], and TADR-EAODV [7] in network simulation experiments. The simulation experiments demonstrate the superiority of the proposed scheme over existing protocols.

## 2. Related Work

Trust models have been widely used in WSNs, but the research on trust models suitable for UWSNs is still relatively small. From the existing research results, trust models can be used in areas such as malicious node detection [8], data collection security [9], and intrusion detection [10].

Yang et al. [7] proposed a trust-aware dynamic routing algorithm augmented with an AODV protocol (TADR-EAODV) protocol for secure communication in WSNs. It considers the trustworthiness of nodes while making routing decisions. It dynamically adjusts the routing paths based on real-time network conditions such as node availability, energy levels, and security factors. Improvements are made in detecting defective nodes and average packet transmission rate. GEDAR is geographic and opportunistic routing protocol. It selects those nodes with the highest Expected Packet Advancement (EPA) as forwarders. The GEDAR protocol solves void routing by adjusting the depth of void nodes. However, node movement may not always be optimal and may consume more energy. Li H et al. [11] proposed a cluster-based vector-based forwarding (CVBF) technique to improve the effectiveness of vector-based forwarding protocols (VBFs) [12]. The entire UWSN volume is divided into multiple rectangles to make clusters, and then a virtual receiver is assigned to each cluster in CVBF. Nicolas et al. [5] extended VBF to the HH-VBF protocol (hop-by-hop VBF protocol). HH-VBF changes the direction of the forwarding pipe by hopping hop-by-hop throughout the lifecycle. Although network performance can be improved by dynamically changing the direction of the flooding pipe compared to VBF, HH-VBF has lower performance in sparse networks when a smaller pipe radius is set. Nandyala et al. [13] proposed a Q-learning-based dynamic network topology aware routing algorithm (QTAR) which uses implicit tangent vertex identification to optimize the selection of the NF (next forwarder), thereby mitigating the energy wastage incurred in forwarding packets away from the receivers and improving network performance. Ganeriwald et al. [14] proposed a trust model RFSN for WSN. In RFSNs, each node monitors the communication records of other nodes and maintains the trust of other nodes locally. Feng et al. [15] proposed a trust model which defined seven categories of trust evidence. However, it is difficult to assign reasonable weights to all trust indicators when fusing multiple pieces of evidence. Ren et al. [16] proposed an unattended WSN trust model based on subjective logic. However, their main focus was ensuring the security of observational evidence storage, so they did not deal with trust assessment and trust update. Han et al. [17] proposed a trust model called Attack-Resistant Trust Model based on Multidimensional trust metrics (ARTMM) in UWSNs. This scheme can reduce the impact of the underwater environment to some extent. However, ARTMM ignores the effect on the network layer after an attack, and it is difficult to accurately describe the trust relationship using only fuzzy logic. Vani et al. [18] used fuzzy logic and cryptographic encryption schemes considering relative mobility, energy, and distance metrics to select secure cluster head nodes and transmit data within the cluster, but their transmission did not consider the energy efficiency and low latency of the forwarding path. He et al. [19] proposed a trust model based on reinforcement learning for UWSNs. The scheme reduces the influence of environment on the trust model to a certain extent and utilizes the reinforcement learning theory to update the trust dynamically, which can cope with changing attack patterns to a certain extent.

Transmission security models for UWSNs mainly rely on the introduction of cryptographic algorithms and routing protocols that are unable to cope with attacks from within the network. The introduction of a trust model to regulate internal nodes is an effective way to deal with internal attacks. Although the existing trust model has made progress in the detection of MNs, it still needs to consider the impact of harsh underwater environments on packet transmission. Therefore, the proposed scheme designed in this paper incorporates intelligent routing with the trust model to find more efficient transmission paths.

## 3. Network Structure

In this section, the UWSN model required for implementation of the proposed algorithm is presented along with some assumptions. The underwater nodes of the UWSNs are randomly distributed within a predetermined range, while the sink nodes (BN) are distributed at the center of the water surface and have no energy constraints to forward the packets from the UW-sensors to the base station.

The UW-sensors are deployed with moorings that stop moving after a certain distance with the current, and their relative positions throughout the individual network structure are relatively stable. Each UW-sensor has acoustic–electrical communication equipment for collecting packets from the seafloor and transmitting the packets to the surface buoy node, and the BPSK [20] modulation technique modulates and demodulates the signal. Each underwater node periodically obtains the position of its neighboring nodes through a position algorithm [21] and maintains a node adjacency list locally; this communication record and status about the neighboring nodes help in choosing the NF. In addition, the UW-sensor maintains a local communication log table with neighboring nodes to continuously record the communication status and history with neighboring nodes.

## 4. Proposed Scheme

### 4.1. The Scheme Overview

**Figure 1:**
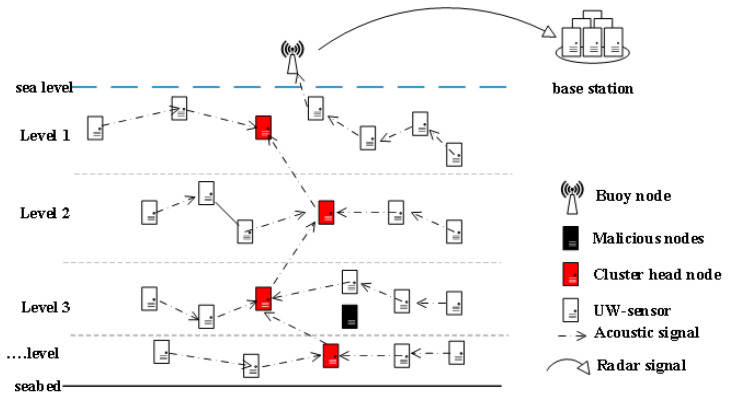
Proposed scheme framework.

This section mainly introduces the model of the proposed scheme. As shown in Figure 1, the BNs are distributed at sea level, the UWSNs are clustered and layered according to the depth of the UW-sensors, the UW-sensor within each time transmits the packets to the cluster head node (CH) inside the cluster, and the CH in each layer accepts the data sent by other UW-sensors and then forwards the packet to the previous CH until it reaches the BN. The non-CH common nodes in the first layer near the BN node can directly transmit data to the BN. The underwater nodes transmit data to avoid the MN detected by the trust model to ensure data security. After calculating the trust value of UW-sensor, the UWSNs will exclude MNs from being pre-selected nodes for data transmission. Before data transmission we need to perform a two-hop availability check on the neighboring nodes to find the void nodes. After removing the void nodes, a path with a smaller total wiring distance is constructed from the remaining neighbor nodes for transmission.

### 4.2. UWSN Clustering

The UWSNs are dynamically clustered according to the depth and distribution of the nodes, as follows:

(1) $$M_{i}=\frac{1+h_{i}}{R_{max}},$$

where the number of UW-sensors in the $k_{th}$ layer is given by:

(2) $$Num_{k}<\frac{node_{num}}{R_{max}},$$

where $node_{num}$ is the number of UW-sensors in the UWSNs, $Num_{k}$ is the number of nodes in the $k_{th}$ layer, i is the node $n_{i},$ $M_{i}$ is the number of layers, $h_{i}$ is the depth of the node $n_{i}$, and $R_{max}$ is the maximum distance of communication.

### 4.3. Election of CH

#### 4.3.1. Input Parameters

In this paper, we used a fuzzy logic algorithm to choose CH, and the trustworthiness of the selected cluster head nodes was mainly judged by three indicators: energy indicator, distance indicator, and node density. The energy indicator ensures that the elected nodes can support the data transmission task of CH, and the distance indicator ensures that the elected CH nodes remain upstream in the cluster and can try to ensure the performance of the UWSNs in terms of packet transmission, as well as reducing energy consumption. The node densities enable better transmission of packet data towards the CH during data transmission.

The formula for calculating node density is shown in Equation (3):

(3) $$V_{ki}=\frac{\left(Neighbor_{iM}\right)}{\left(Num_{M_{i}}\right)},$$

where $Neighbor_{iM}$ is the number of neighboring nodes of UW-sensor $n_{i}$ in layer M. $Neighbor_{iM}$ satisfies two conditions: the first is that the neighboring nodes belong to the same layer M as node $n_{i}$, and the second is that the distance between the neighboring node and $n_{i}$ is less than $0.9R_{max}$. $Num_{M_{i}}$ is the number of nodes in layer *M*.

The energy metrics are given by Equation (4), where $E_{res}^{i}$ is the residual energy of the UW-sensor $n_{i}$, and $E_{initia}^{i}$ is the initial energy of the UW-sensor $n_{i}$, which is the same for all UW-sensors.

(4) $$e_{i}=\frac{\left(E_{res}^{i}\right)}{\left(E_{initia}^{i}\right)},$$

(5) $$d_{i}=\frac{1-d_{iB}}{d_{max}},$$

where $d_{iB}$ is the distance from the UW-sensor $n_{i}$ to the BN, and $d_{max}$ is the maximum distance of the network among all nodes to the surface BN. The larger the node of the distance indicator $d_{i}$, the closer to the core area in the node network.

#### 4.3.2. Fuzzy Algorithms

**Figure 2:**
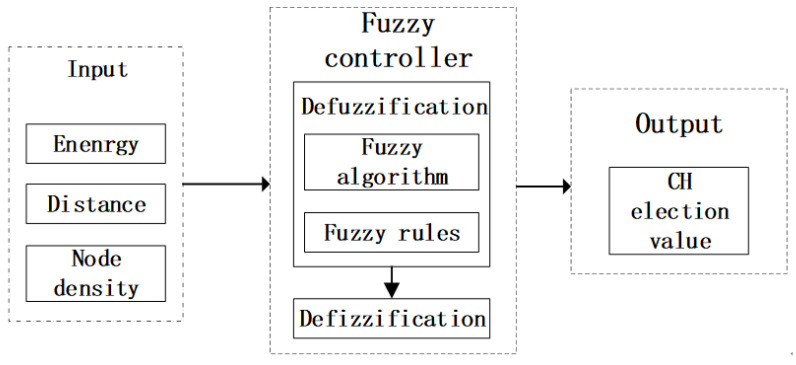
Flowchart of the fuzzy algorithm.

The use of fuzzy algorithms [22] is able to better solve fuzzy, difficult-to-quantify problems by mimicking human experience and decision-making behavior to arrive at a simple and effective overall evaluation of the object or physical object, so in this paper study we used a fuzzy algorithm to evaluate three indicators for each node in the cluster to determine the node that is more suitable to take on the role of CH. As shown in Figure 2, the algorithm is divided into three parts: first of all, we have to input three fuzzy variables into the fuzzy controller; in the fuzzy controller the fuzzy variables will be fuzzy, through the fuzzy algorithm as well as the fuzzy rules to obtain the fuzzy evaluation of the things; and then the fuzzy evaluation is de-fuzzified to obtain an accurate value.

The fuzzification process of fuzzy algorithms requires the conversion of clear values of fuzzy parameters into fuzzy values. The membership functions of the fuzzy linguistic variables of the input fuzzy parameters obey the trapezoidal affiliation function. The fuzzy inference module needs to be utilized to make the conversion, and these conversion rules are called fuzzy rules. The fuzzy rules defined by the proposed scheme are based on If–Then statements and the fuzzy AND operation and OR operation.

For example: IF (distance 0.75 to 1) and (energy 0.8 to 1) and (node density 0.75 to 1) THEN (total trust is high).

We make use of the characteristics of the fuzzy algorithm and the clear input values to determine the fuzzy rules and thresholds in Table 1 according to the experience of other scholars [23].

The rules for the proposed scheme using the fuzzy rules are specifically shown in Table 1.

The membership function [24] is essentially a curve that represents the way membership is assigned to a specified clear multi-attribute trust value. There are different types of membership functions, and the trapezoidal function [25] is less computationally complex if it is accurate enough.

#### 4.3.3. Defuzzification

To get a clear overall rating of a CH election node, the fuzzy results obtained from the fuzzification process are defuzzified to derive the exact value. The objective algorithm uses the center of mass method for defuzzification to calculate the node ratings and selects the highest election value in the cluster as the CH, as shown in Equation (6).

(6) $$T_{CH}=\frac{\Sigma_{k=1}^{m}\nu_{k}\mu_{\nu }(\nu_{k})}{\Sigma_{k=1}^{m}\mu_{\nu }(\nu_{k})},$$

where $\nu_{k}$ and $\mu_{\nu }(\nu_{k})$ denote the area of the subregion and the center-of-mass partition of area $\nu_{k}$, respectively.

### 4.4. Trust Model

To identify MNs in UW-sensors, the proposed solution calculates the selection of trust metrics in the communication interactions of UW-sensors as well as in the monitoring structure by constructing a trust model, and the trust metrics are further processed and transformed into three trust metrics through the trust model: direct trust, indirect trust, and environmental trust. The trust metrics of the nodes are calculated and updated within a time window. The calculation of trust can exclude MNs from the UWSNs and ensure the security of packet transmission.

#### 4.4.1. Direct Trust

The direct trust proposed by the proposed scheme calculates three main trust metrics: energy trust, historical interaction records, and data trust. When selecting forwarding nodes, the energy of forwarding nodes is an important evaluation metric, and insufficient energy cannot guarantee that forwarding nodes can reliably transmit data packets to receiving nodes. Evaluating the historical interaction records between nodes can effectively identify malicious behavior within the network, judge the interaction behavior of nodes, and identify MNs. Computing data trust can check the consistency and fault tolerance of generated or forwarded data within the underwater nodes and then identify the MNs that tamper with the data or modify the data.

Energy trust: In addition to being an important indicator to ensure reliable data transmission, energy trust can also be used as a monitoring indicator to identify MNs. For example, MNs will consume extra energy of the nodes when they perform man-in-the-middle attacks. Especially when they perform DOS attacks, MNs send a large number of redundant messages, which consume the energy of the other nodes in the UWSNs and occupy the channel. So, the computation of energy trust can identify MNs in the UW-sensors in time to detect DOS and other malicious node attacks [26]. The proposed scheme defines the energy trust as the ratio of the average energy of the next hop node to the neighboring nodes, as follows:

(7) $$T_{energy}=\frac{e_{res}^{i}}{e_{avg}^{i,nei}}$$

where $e_{res}^{i}$ is the residual energy of UW-sensor $n_{i}$ and $e_{avg}^{i,nei}$ is the average residual energy of neighboring nodes of node $n_{i}$.

Historical interaction trust: Underwater nodes obtain historical interaction records with neighboring nodes by accessing the locally maintained communication log table and determine the behavior of the node through these records to calculate the historical interaction trust. This can effectively identify the malicious behavior of MNs, such as gray hole attacks, which are switching attacks in which MNs maliciously drop packets through a certain time period or selectively, affecting the normal communication of the network and destroying the availability as well as the accuracy of the transmitted data. The proposed scenario defines the historical interaction trust as follows:

(8) $$T_{history}=\frac{N_{sr}^{j}}{N_{tr}^{jnei}}+\frac{N_{ss}^{j}}{N_{ts}^{jnei}},$$

where $N_{sr}^{j}$ and $N_{ss}^{j}$ are the number of packets received and sent by the next hop node $n_{i}$ to the source node and $N_{tr}^{jnei}\;and\;N_{ts}^{jnei}$ are the number of packets received and sent by node $n_{i}$ to neighboring nodes.

Data trust: The main task of UWSNs is to transmit data. To prevent MNs from tampering or fabricating false data, the proposed scheme evaluates the trustworthiness of data transmission, according to the research of previous scholars [27]. Theoretically, the data received by neighboring underwater sensor nodes for a certain thing are similar, showing certain spatiotemporal correlation, and these theoretical data obey normal distribution. However, there is a certain difference between the actual value and the theoretical value, so the proposed scheme can effectively solve this problem by appropriately expanding the error threshold. The data trust for CH is calculated based on a set of data received in the previous time window, and the data trust is updated in the clustering for this time window. The proposed scheme defines the data trust specifically, as shown in Equations (9) and (10):

(9) $$f(x)=\frac{1}{\sigma \sqrt{2\pi }}e^{\left(-\frac{(x-\mu {)}^{2}}{2\sigma^{2}}\right)},$$

where $x_{i}$ is the value of the data item of the monitored underwater node sensor and μ and σ are the mean and variance of the data, respectively.

After calculating the formula for the normal distribution of the data, the data trust is calculated as shown in Equation (10):

(10) $$T_{data}=2\left(0.5-|\int_{\mu }^{x.}\;f(x)dx|\right),$$

Direct trust: The proposed scheme fuses several of the trust metrics mentioned above and calculates the direct trust of the pre-selected next-hop node, as shown in Equation (11):

(11) $$T_{direct}=\omega_{1}\times T_{energy}+\omega_{2}\times T_{history}+\omega_{3}\times T_{data},$$

where $\omega_{1}$, $\omega_{2}$, and $\omega_{3}$ are used to calculate the weight of trust during the initialization process in embedding.

#### 4.4.2. Indirect Trust

Indirect trust is also an important metric for evaluating a node, and it is necessary to use indirect trust to evaluate a node when direct trust cannot accurately calculate the reliability of a node. Since there may be MNs in the neighboring nodes to make malicious recommendations to the pre-selected next-hop nodes, it is necessary to evaluate the reliability of the recommendations to exclude malicious recommendations.

If the source UW-sensor needs to obtain the indirect trust of the pre-selected UW-sensor, it sends a request to a set of public neighbor nodes and selects a set of indirect trust recommendation groups in the group of public neighbor nodes whose direct trust is greater than 0.5. The nodes in the recommendation trust group are not completely reliable, and it is still necessary to test the reliability of their recommendation of indirect trust to exclude the outliers and calculate the indirect trust. This effectively solves the problem of false positives of malicious nodes.

For reliability assessment of indirect trust, the Gaussian distribution anomaly detection algorithm is utilized. For a set of recommended trust values $R=(R_{1},R_{2},R_{3},\ldots ,R_{m})$, the outliers are identified, and the reliability is computed as shown in Equations (12) and (13):

(12) $$\rho (T)=\frac{1}{\sigma \sqrt{2\pi }}e^{\left(-\frac{(T-\mu {)}^{2}}{2\sigma^{2}}\right)}$$

$where\;T_{i}$ is the trust value recommended by UW-sensors in the recommended trust group, and μ and σ are the mean and variance of the recommended trust values in this group, respectively. For judging abnormal recommended trust values, the proposed scheme is defined as the value whose value ${\;T}_{i}$ deviates from the mean by more than two times the standard deviation. $T_{i}$ will be considered as an abnormal value and will not participate in the indirect trust calculation, as follows:

(13) P(|T−μ|>2σ)≤0.477

The reliability of the recommended trust value after excluding outliers is given by:

(14) $$T_{rec}^{in}=1-2|\int_{\mu }^{R}\;f(R)dR|$$

where f(R) is the trust value distribution function of Equation (12). For a set of recommended trust values $R=(R_{1},R_{2},R_{3},\ldots ,R_{C})$ that excludes outliers, the reliability $T_{rec}^{in}$ is obtained for each of these trust values, and the indirect trust calculation formula is given by:

(15) $$T_{indirect}=\frac{\sum_{i=1}^{c}\;T_{rec}^{i}\times R_{i}}{c}$$

where c is the number of recommended UW-sensors participating in the computation, $R_{i}$ is the recommended trust value of node $n_{i}\;$ to the pre-selected nodes, and $T_{rec}^{i}$ is the assessment of the reliability of the recommended trust value of node $n_{i}$.

Environmental trust: Hydroacoustic communication will be affected by a variety of factors. For example, environmental noise will lead to poor quality of the communication link, resulting in fluctuations in communication, energy, and data, which in turn leads to miscalculation of the trust between the nodes and reduces the accuracy of the detection for MN.

Using the underwater acoustic signal path attenuation model [28], the absorption coefficient α(f) estimated from Thorp’s formula is given by:

(16) $$\alpha (f{)}_{=}\frac{0.11f^{2}}{1+f^{2}}+\frac{44f^{2}}{4100+f^{2}}+2.75\times 10^{-4}f^{2}+0.003$$

where f is the signal frequency. The average signal-to-noise ratio is given by:

(17) $$SNR(d,f)=\frac{P/(d^{k}a(f{)}^{d})}{N(f)B}$$

where k is the spreading factor in the noise simulation, which represents the propagation loss and is set to 1.7 for practical propagation, d is the distance, B is the bandwidth, and N(f) is the PSD of noise in acoustic communication, including four categories: turbulence noise, ship noise, wave noise, and thermal noise [29]. In the 1 < *f* < 100 KHz range, the PSDs of the noise categories are very close to each other, and the estimation equation is given by:

(18) $$N(f)\approx N_{1}-\tau 10log(f)$$

where $N_{1}$ and τ are experimentally obtained constants. Based on the calculation of Equations (16)–(18) above, the BER calculation for conditional channel transmission at frequency *f* and distance *d* is given by:

(19) $$LT_{ij}=1-p.(d,f)$$

#### 4.4.3. Total Trust

Total trust in the proposed scheme is calculated using three indicators: direct trust, indirect trust, and environmental trust:

(20) $$T_{total}=\lambda T_{direct}+\beta T_{indirect}+\gamma LT_{ij}$$

where β, λ, and γ are constants between 0 and 1, and add up to 1, and β, λ, and γ are the weight of the total trust calculated.

Trust update: In the trust model of the proposed scenario, to reflect the state of UWSNs in a timely manner and maintain the timeliness of the trust values, each UW-sensor of the UWSNs performs the trust value computation as well as the trust update at the beginning of each time window and uses the new trust value as an important basis for interaction with other nodes.

At the beginning of each time window, the trust of the previous time window is used as the historical trust with the calculated trust of the current window for trust update. However, the trust values are time-sensitive, and the latest trust values are much more important than the historical trust, so the historical trust decays exponentially [30], and the trust update calculation formula is given by:

(21) $$T_{t+1}=T_{t+1}^{cur}+\frac{T_{t}}{W_{t}}$$

where $T_{t+1}^{cur}$ is the current trust, $T_{t}$ is the trust of the previous round, and $W_{t}$ is the time decay factor that needs to be used for specific underwater scenarios.

### 4.5. Data Forwarding

At the beginning of the time window, the trust model of the proposed scheme will allow each UW-sensor to record the performance of the neighboring UW-sensor, as well as the channel condition. After calculating the trust value of the UW-sensors, the UWSNs will exclude UW-sensors with trust values below 0.5 from being pre-selected nodes for data transmission. An explanation as to why the threshold is chosen to be 0.5 will be given in Section 5, Simulation and Analysis of Results. Because of the limitations of hydroacoustic communication, most of the energy of UW-sensors is consumed in sending and receiving datagrams, so to reduce the communication consumption of UW-sensors, the proposed scheme will prioritize the transmission routes in the set of candidate neighbors $Neighbor_{i}$ to choose the transmission paths taking into account the total cabling distance of the CHs or BNs. But this leads to possible routing voids in the network [31]. To solve the routing voids, before data transmission we need to perform a two-hop availability check on the neighboring nodes to find out the void nodes. After removing the void nodes, a path with a smaller total wiring distance is constructed from the remaining neighbor nodes for transmission. The algorithm for the data forwarding phase is shown in Algorithm 1:
**Algorithm 1:** Packet forwarding
$\mathrm{Procedure}\;\mathrm{forwarding}\;\mathrm{data}\;(\mathrm{node}\;n_{i}$)
Initialize;
Get ID of CH, locations of Neighbor; locations of CH, BN
Calculate the trust value of neighboring nodes 
$\mathrm{For}\;n_{j}$ in network do
$\mathrm{If}\;(n_{j}<0.5$)
 $\mathrm{Exclude}\;n_{j}$ from the network;
End if
End for
$\mathrm{If}\;(\mathrm{the}\;\mathrm{distance}\;\mathrm{from}\;n_{i}\;to\;BN<R_{max}$)
$n_{i}$ next hop is BN;
End if
$\mathrm{If}\;(n_{i}$ $!=\mathrm{CH}\;\mathrm{and}\;\mathrm{the}\;\mathrm{distance}\;\mathrm{from}\;n_{i}\;to\;BN>R_{max}$)
   ${Neighbor}_{i}$$\leftarrow \mathrm{two}\;\mathrm{hop}\;\mathrm{check}\;(n_{i}$);
End if
$\mathrm{For}\;n_{j}$ $\mathrm{in}\;{Neighbor}_{i}$ do
$\mathrm{Calculate}\;\mathrm{the}\;\mathrm{distance}\;\mathrm{from}\;n_{j}$ to CH;
$\mathrm{Calculate}\;\mathrm{the}\;\mathrm{distance}\;\mathrm{from}\;n_{i}$ $\mathrm{to}\;n_{j}$;
Calculate the total distance;
$\mathrm{Check}\;\mathrm{the}\;n_{j}$ $\mathrm{with}\;\mathrm{the}\;\mathrm{smallest}\;\mathrm{total}\;\mathrm{distance}\;\mathrm{in}\;{Neighbor}_{i}$;
$\mathrm{Set}\;a\;\mathrm{timer}\;\mathrm{for}\;n_{j}$;
Sending packets
Update the rate of communication record table
End for
//supplement
$\mathrm{While}\;\mathrm{the}\;\mathrm{timer}\;\mathrm{for}\;n_{j}$ is expired
$\mathrm{If}\;\mathrm{do}\;\mathrm{not}\;\mathrm{get}\;\mathrm{ACK}\;\mathrm{from}\;n_{j}$
$\mathrm{Sending}\;\mathrm{packets}\;\mathrm{to}\;\mathrm{next}\;\mathrm{node}\;\mathrm{in}\;{Neighbor}_{i}$
End if
End while
End procedure

Initialization: when the network model is initialized, the network will initialize the energy, neighbor table, and local communication record table, obtain the remaining energy, location information, and data trust from the previous time window, and obtain other information to cluster the network. The cluster head node will be re-elected at the beginning of each time window, and the ID, as well as the location information of the CH, will be broadcast in the network. The normal underwater nodes will only receive the CH information of the corresponding layer, as well as the information of the BN.

Filtering the candidate set: after the trust model excludes the MNs below the threshold from the network, the underwater node $n_{i}$ checks whether the BN node is within its communication range or not, because the nodes close to the BN nodes in the first layer of the cluster are more likely to be the CH and have to take on more data transmission tasks, so to alleviate the energy consumption of the UW-sensors in this area, these UW-sensors do not transmit packets to the CHs in the first layer. Instead, they transmit data directly to the BN. The other underwater nodes will transmit data to the CH in the same layer. To improve the performance of the UWSNs, the algorithm prioritizes the total wiring distance, and to solve the possible routing void problem, a two-hop availability check is performed on the neighboring nodes to find void nodes before the data transmission. The algorithm finds a node in the neighbor table that is closer to the CH than the source node and checks if this neighbor node has a neighbor node that is closer to the CH and if the neighbor node is a void node.

**Figure 3:**
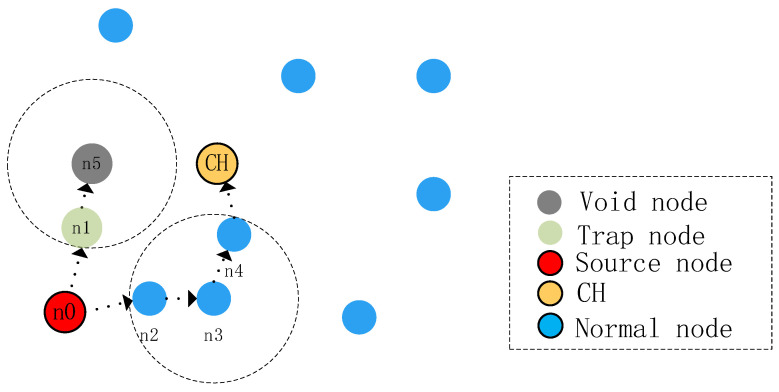
Example of two-hop availability check.

For two-hop availability checking, as shown in Figure 3, when node n0 of the data sends a packet, there are two legitimate nodes $n_{1}$ and $n_{2}$ that can transmit it, and then, according to the routing protocol, we check the neighbor table of the $n_{1}\;and\;n_{2}$ nodes and find that both nodes have legitimate forwarding nodes $n_{3}$ and $n_{5}$. Then, when we query the neighbor table of the $n_{5}$ node, we find that there is no adjacent node of node $n_{5}$ that can transmit the data to the CH; the protocol then marks $n_{5}$ as a void node and $n_{1}$ as a trap node, excluding $n_{5}$ as well as $n_{1}$ from the candidate set during data transmission.

After removing the void node, the source node n0 will sort the nodes in the candidate set for sending according to the total wiring distance, but because of the variable underwater environment and high complexity, there may be a situation where data transmission to the forwarding node with high priority fails at a certain time, and if it does not select the next priority node as a forwarding node in a timely manner, there will be a phenomenon that consumes energy, occupies bandwidth, and reduces data delivery rate. So, we have designed a supplement to the routing algorithm of the scheme in the supplemental algorithm. We set a timer for the forwarding node. When the priority node expires at its timer time and the source node n0 has not received the ACK message returned by the priority node (which indicates that the packet has been accepted and forwarded to the next node), the source node n0 forwards the message to the next priority node until the data arrive at the CH or BN.

## 5. Simulation

Simulations were performed using the proposed scheme by MATLAB (R2022a). We conduct a comprehensive comparison of our scheme with the other schemes: HH-VBF, GEDAR, and TADR-EAODV. To prove the accuracy of the experiments we simulated the target scheme with the HH-VBF, GEDAR, and TADR-EAODV schemes under the same network conditions.

### 5.1. Simulation Setting

We simulated 50–100 underwater node deployments with a communication range set to 700 m. The UW-sensors in the underwater network are randomly distributed, all nodes can generate data packets, and a sink node is distributed in the central water surface of the monitoring area The specific simulation settings are shown in Table 2.

We simulated three typical attacks on internal nodes: black hole attack, gray hole attack, and DOS. In a black hole attack, the infected MNs will refuse to accept and forward any packet, affecting data communication. The malicious node in a gray hole attack will discard the accepted packets randomly; the packet discard rate of the gray hole attack set for the experiments in this paper was 0.4. The malicious node in the DOS attack simulated in this paper will forward a large number of duplicate packets or send false packets, which occupies the channel resources of the network and makes normal nodes unable to communicate. In addition to this, to verify the network performance as well as the reliability of the proposed algorithm, the experiment also evaluated the average energy consumption (AEC), average end-to-end delay (AE2ED), and other aspects of the UWSNs.

### 5.2. Results

**Figure 4:**
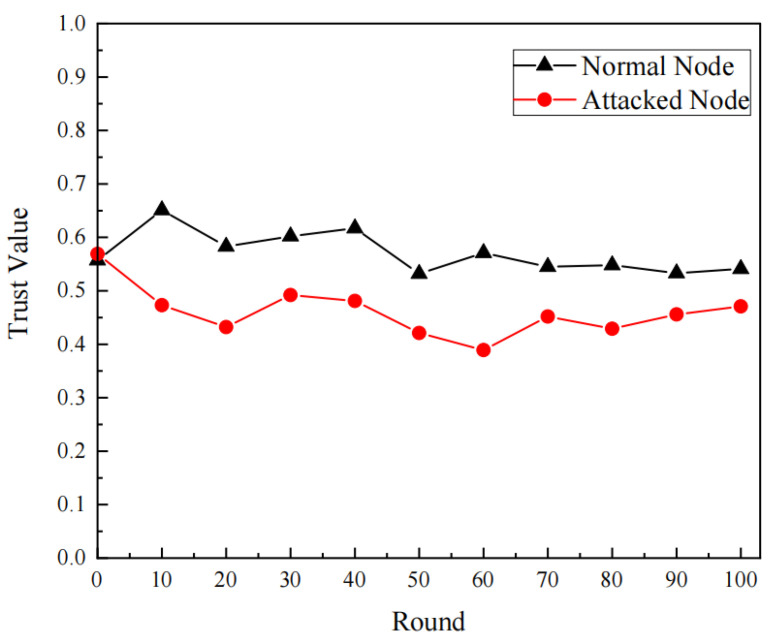
Average trust values of nodes during simulated attack with respect to rounds.

As shown in Figure 4, regarding the change of the average trust of normal UW-sensors and MNs in the UWSNs during the attack between 0–100 rounds, the average trust of the normal UW-sensors of the proposed scenario fluctuated between 0.55 and 0.65, and the MNs’ average trust value of the UW-sensors stabilized between 0.4 and 0.5 after 10 rounds of updating the trust value, so in the previous section setting the MN detection of the threshold set to 0.5 was accurate. The threshold of 0.5 is the data point obtained from the simulation experiment. Because the scheme calculates the impact of the environment on data transmission, the threshold of 0.5 depends on the network and is not fixed.

As shown in Table 3, to test the detection accuracy of the proposed scheme for MN, the simulation experiment was set up with 100 underwater nodes, 150 rounds, and ratios of MN from 10% to 30%. The simulation experiment shows that the increase in the ratio of the MNs for the proposed scheme puts a greater burden on the detection accuracy of the proposed scheme. The detection rate of MNs decreased slowly with the ratio of MNs, but the reduction in MN detection rate was relatively small, indicating that the trust model used by the proposed scheme has strong reliability for the detection of MN, as well as the ability to resist stress.

As shown in Table 4, to detect changes in the detection accuracy of the trust model proposed by our scheme for MNs according to the number of rounds, the simulation experiment was set to 100 underwater nodes, 100–300 rounds, and a rate of 20% of MNs in the underwater nodes. The simulation experiments showed that the detection rate of MNs increases with the increase in UWSN operation time. This is because the proposed scheme detects the MNs and quickly isolates them from the UWSNs to mitigate the impact of MNs on the UWSNs. With the increase in simulation rounds, the model will evaluate the MNs in the network more accurately, indicating that the trust model used by the proposed scheme is more reliable in evaluating MNs.

**Figure 5:**
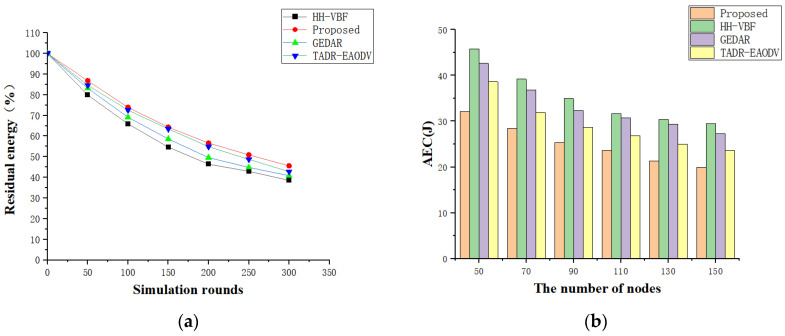
(**a**) Residual energy vs. number of turns; (**b**) AEC vs. number of nodes.

As shown in Figure 5a, regarding the simulation results of energy consumption (the average remaining energy of the network node) versus the UWSN operation time when the simulation experiment has 100 underwater nodes, the proposed scheme has a relatively low energy consumption compared to the other schemes as the increase in UWSN operation time. Figure 5b shows the results of AEC per round versus the number of nodes. Combining Figure 5a with Figure 5b shows that the proposed scheme’s energy consumption is significantly lower as compared to the HHVBF and GEDAR protocols. Because the proposed scheme has screened out a number of MNs or defective nodes with very poor communication environments during the trust calculation, it prioritizes the total cabling distance in data transmission and avoids the void areas. This makes the proposed scheme more energy-efficient.

**Figure 6:**
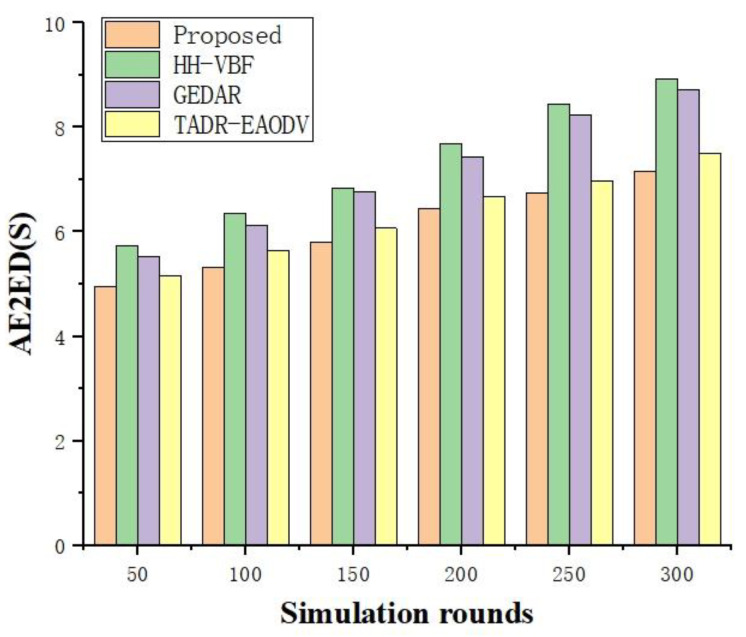
AE2ED vs. rounds.

As shown in Figure 6, as the number of simulation rounds increases, the AE2ED also increases. This is mainly due to the fact that, as the UWSN operates, some of the UW-sensors consume energy too quickly, resulting in data forwarding paths having to choose nodes with longer total cabling distances. The AE2ED of the scheme is always lower than the other three schemes, while HH-VBF and GEDAR have the worst performance because these two algorithms are limited to nodes within the radius of the pipeline or nodes shallower than the current node, which is not flexible enough in forwarding node selection, resulting in their higher AE2EDs.

**Figure 7:**
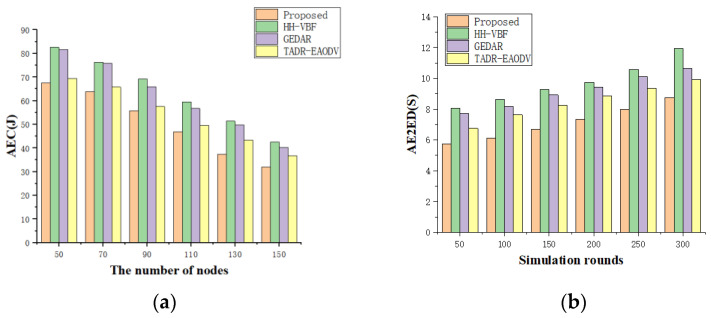
(**a**) AEC vs. number of nodes (20 MN); (**b**) AE2ED vs. rounds (20 MN).

As shown in Figure 7a, 20 malicious nodes were randomly distributed in the setting node of the simulation experiment, and the simulation results of the average energy consumption (AEC) per round of the network and the number of nodes were obtained. As can be seen from the figure, the energy consumption of the target scheme was relatively low compared with other schemes. Figure 7b shows the random distribution of 20 malicious nodes in the setting node of the simulation, and the results of the AE2ED and simulation rounds. By combining Figure 7a,b, compared with the other three protocols, when there are malicious nodes in the network, the network performance of the target scheme is better than that of the other three protocols, and the impact of malicious nodes on the network is less than that of the other two protocols: HH-VBF and GEDAR perform the worst. This is because they lack security mechanisms to respond effectively to malicious nodes. This proves that the trust model can effectively identify malicious nodes, and the target scheme is robust enough to detect malicious nodes.

## 6. Conclusions

Due to the many limitations of the environment in which underwater sensor networks are located, underwater sensor networks are unsupervised environments, and the possibility of data exposure or attack by MNs in the UWSNs is high. To ensure secure packet transmission in underwater sensor networks while maximizing the efficiency of packet transmission, we proposed to come up with a routing protocol for UWSNs based on a trust model and void avoidance algorithm. The trust model was used to detect MNs within the UWSNs, and environmental trust was considered to exclude some defective nodes with very poor communication environments from the routing. In addition, the total wiring distance of the route and the null node avoidance algorithm were also considered to create as much as possible an efficient and reliable transmission route. The simulation results were compared with several schemes (HH-VBF, GEDAR, and TADR-EAODV). The target scheme has excellent performance and high accuracy, as well as reliability for malicious node detection.

## Figures and Tables

**Table 1:** Fuzzy rules.

Rule Number	Inputs			Output
	Distance	Energy	Neighborhood Density	Rating Levels
1	0.75 to 1.0	0.8 to 1.0	0.75 to 1.0	High
2	0.75 to 1.0	0.4 to 0.8	0.39 to 0.75	High
3	0.75 to 1.0	0 to 0.39	0 to 0.4	Low
4	0.75 to 1.0	0.8 to 1.0	0.39 to 0.75	High
5	0.75 to 1.0	0.4 to 0.79	0 to 0.4	Medium
6	0.75 to 1.0	0.4 to 0.79	0.75 to 1.0	High
7	0.75 to 1.0	0.8 to 1.0	0 to 0.4	Medium
8	0.75 to 1.0	0 to 0.39	0.8 to 1.0	Low
9	0.75 to 1.0	0 to 0.39	0.39 to 0.75	Low
10	0.4 to 0.74	0.8 to 1.0	0 to 0.4	Medium
11	0.4 to 0.74	0.4 to 0.79	0.75 to 1.0	High
12	0.4 to 0.74	0.4 to 0.79	0 to 0.4	Low
13	0.4 to 0.74	0 to 0.39	0.8 to 1.0	Low
14	0.4 to 0.74	0 to 0.39	0.39 to 0.75	Low
15	0.4 to 0.74	0.8 to 1.0	0.39 to 075	Medium
16	0.4 to 0.74	0 to 0.39	0 to 0.4	Low
17	0.4 to 0.74	0.8 to 1.0	0.8 to 1.0	Medium
18	0.4 to 0.74	0.4 to 0.79	0.39 to 0.75	Medium
19	0 to 0.39	0 to 0.39	0 to 0.4	Low
20	0 to 0.39	0.8 to 1.0	0.39 to 0.75	Medium
21	0 to 0.39	0.8 to 1.0	0 to 0.4	Low
22	0 to 0.39	0.4 to 0.79	0.8 to 1.0	Medium
23	0 to 0.39	0.4 to 0.79	0 to 0.4	Low
24	0 to 0.39	0 to 0.39	0.39 to 0.75	Medium
25	0 to 0.39	0 to 0.39	0.8 to 1.0	Low
26	0 to 0.39	0.8 to 1.0	0.8 to 1.0	Medium
27	0 to 0.39	0.4 to 0.79	0.39 to 0.75	Medium

**Table 2:** Simulation condition settings.

Simulation Condition Settings	Value
Number of nodes	50–150
Space measuring	1500 × 1500 × 1000 m
Initial energy	1000 J
Frequency	20 kHz
Packet size	50–100 bytes
Data rate	4 kbps
Transmission power	10 W
Receiver power	2 W
Communication range	700 m
Malicious node	10–30%
Idle power	15 mW
Sink node	1

**Table 3:** Malicious node detection accuracy with malicious node ratios.

MN Ratio	10%	15%	20%	25%	30%
DOS	0.846	0.862	0.858	0.859	0.853
Black hole attack	0.948	0.956	0.952	0.943	0.935
Gray hole attack	0.852	0.864	0.871	0.864	0.856

**Table 4:** Malicious node detection accuracy with Simulation rounds.

Simulation Rounds	100	150	200	250	300
DOS	0.823	0.858	0.872	0.891	0.907
Black hole attack	0.948	0.952	0.959	0.963	0.975
Gray hole attack	0.852	0.871	0.889	0.906	0.912

## Data Availability

The original contributions presented in this study are included in the article; further inquiries can be directed to the corresponding authors.
